# Density gradient centrifugation specifically improves sperm motility in hyperuricemia: evidence from intrauterine insemination cycles – retrospective cohort study

**DOI:** 10.1186/s12610-025-00292-z

**Published:** 2025-11-07

**Authors:** Jinqiang Peng, Lingjuan Wu, Zhimin Li, Qiongying Huang, Lixian Li

**Affiliations:** Reproductive Medical Center, Affiliated Hospital of Putian University, 999 Dongzhen East Road, Putian, Fujian China

**Keywords:** Hyperuricemia, Male infertility, Sperm motility, Density gradient centrifugation, Reactive oxygen species, Intrauterine insemination, Hyperuricémie, Infertilité masculine, Mobilité des Spermatozoïdes, Centrifugation à Gradient de Densité, Espèces réactives de l’Oxygène, Insémination intra-utérine

## Abstract

**Background:**

Hyperuricemia (HUA) impairs sperm function via oxidative stress and metabolic dysregulation. This retrospective cohort study aimed to investigate the therapeutic effect of density gradient centrifugation (DGC) on HUA-associated sperm dysfunction.

**Results:**

A total of 490 couples undergoing their first intrauterine insemination (IUI) were stratified into the HUA group (200 cycles) and control group (290 cycles) based on male serum uric acid levels. At baseline, the percentage of progressively motile sperm (PR%) in the HUA group was significantly lower than that in the control group (39.55% ± 11.29% vs. 41.76% ± 11.89%, *P* = 0.040). Following DGC processing, PR% in both groups exceeded 90% with no significant intergroup difference; however, the increase in PR% (ΔPR%) was significantly greater in the HUA group (52.34% ± 10.62% vs. 50.29% ± 11.02%, *P* = 0.040). No significant difference was observed in the clinical pregnancy rate between the two groups (11.0% vs. 13.4%, *P* = 0.230).

**Conclusions:**

DGC specifically improves sperm motility in patients with HUA. While direct measurement of mechanistic markers (e.g., oxidative stress, metabolic factors) was not performed in this study, this motility-improving effect may correlate with DGC’s known capacity to scavenge reactive oxygen species and optimize energy supply—an inference supported by prior mechanistic studies. However, improving sperm motility alone is insufficient to significantly enhance clinical pregnancy rates. These findings provide insights to optimize semen preparation strategies in HUA-associated male infertility.

## Résumé

**Contexte:**

L’hyperuricémie altère la fonction des spermatozoïdes via un stress oxydatif et une dérégulation métabolique. Cette étude de cohorte rétrospective visait à étudier l’effet thérapeutique de la centrifugation à gradient de densité sur le dysfonctionnement des spermatozoïdes associé à l’hyperuricémie.

**Résultats:**

Au total, 490 couples bénéficiant de leur première insémination intra-utérine (IIU) ont été classés dans le groupe hyperuricémie (200 cycles) et le groupe témoin (290 cycles) selon les taux sériques d’acide urique masculin. Au départ, le pourcentage de spermatozoïdes à mobilité progressive (SMP %) dans le groupe hyperuricémie était significativement inférieur à celui du groupe témoin (39,6 % ± 11,3 % vs 41,8 % ± 11,9 %, p = 0,040). Après le traitement par centrifugation à gradient de densité, le pourcentage de SMP dans les deux groupes a dépassé 90 %, sans différence significative entre les groupes ; cependant, l’augmentation du pourcentage de SMP (ΔSMP %) était significativement plus importante dans le groupe hyperuricémie (52,3 % ± 10,6 % vs 50,3 % ± 11,0 %, p = 0,040). Aucune différence significative n’a été observée dans les taux de grossesse clinique entre les deux groupes (11,0 % vs 13,4 %, p = 0,230).

**Conclusions:**

La centrifugation à gradient de densité améliore spécifiquement la motilité des spermatozoïdes chez les patients atteints d’hyperuricémie. Bien que la mesure directe des marqueurs mécanistes (par ex., le stress oxydatif, les facteurs métaboliques) n’ait pas été effectuée dans cette étude, cet effet d’amélioration de la motilité peut être corrélé avec la capacité connue de la centrifugation à gradient de densité à piéger les espèces réactives de l’oxygène et à optimiser l’approvisionnement en énergie – une inférence étayée par des études mécanistes antérieures. Cependant, l’amélioration de la mobilité des spermatozoïdes ne suffit pas à elle seule à améliorer les taux de grossesse clinique. Ces résultats fournissent des informations pour optimiser les stratégies de préparation du sperme dans l’infertilité masculine associée à l’hyperuricémie.

## Introduction

In recent years, the incidence of hyperuricemia (HUA) has risen annually alongside significant changes in the dietary patterns, lifestyles, and environments of the global population. An epidemiological study of HUA in China demonstrated that from 2001 to 2017, the prevalence of HUA in China increased from 8.5% to 18.4% [1]. HUA not only causes gout but also impairs male fertility through multiple mechanisms, including oxidative stress, metabolic dysregulation, and endocrine disorders [2–6]. In the field of assisted reproductive technology (ART), density gradient centrifugation (DGC) has emerged as one of the most widely used methods for semen processing prior to intrauterine insemination (IUI) due to its capacity to efficiently screen for high-motility sperm [7, 8]. However, existing studies have focused primarily on the comparison of DGC with other semen processing methods and evaluations of DGC efficacy in the general infertile population [9–13]; studies on patients with HUA, a unique group with metabolic abnormalities, are lacking. This knowledge gap has resulted in a paucity of personalized semen management protocols for patients with HUA, which may impact pregnancy outcomes in those undergoing IUI. This study aimed to address two core objectives: (1) To verify whether HUA impairs sperm motility by exacerbating oxidative stress, using baseline semen parameter differences between HUA and normouricemic controls; (2) To evaluate whether DGC can specifically reverse HUA-associated sperm motility damage, and whether this effect correlates with DGC’s capacity to reduce oxidative stress. Clinical pregnancy outcomes were also compared to clarify the clinical relevance of these findings.

## Patients and methods

### Study participant and grouping

Clinical data were collected from 490 couples undergoing their first IUI cycle at our center between April 2017 and February 2025. Inclusion criteria were as follows: (1) Couples with male infertility or unexplained infertility; (2) Male partners undergoing their first IUI cycle; and (3) Couples enrolled in a natural cycle protocol (defined as IUI cycles without ovulation-inducing medications: follicular development and ovulation were monitored via transvaginal ultrasound, confirmed by a luteinizing hormone surge, with IUI performed 24–36 h after ovulation confirmation).

The exclusion criteria were as follows: (1) Genetic or congenital abnormalities, endocrine abnormalities, varicocele, infectious diseases, systemic diseases, and immune abnormalities; (2) Fever in the past 3 months or exposure to high-temperature environments, chemicals, heavy metals, and other adverse factors that hinder spermatogenesis; and (3) A history of testicular injury, a history of mumps, or long-term medication use.

The definitions of the study groups were as follows: According to the serum uric acid (SUA) level of the male partners, the participants were stratified into two groups: the control group (SUA level ≤ 420 µmol/L) and the HUA group (SUA level > 420 µmol/L). This threshold was determined based on domestic authoritative clinical guidelines: *Guideline for the Diagnosis and Management of Hyperuricemia and Gout in China (2019)* [14] and *China Multi-Disciplinary Expert Consensus on Diagnosis and Treatment of Hyperuricemia and Related Diseases (2023 Edition)* [15]. It is also consistent with the grouping criteria used in reproductive medicine studies investigating HUA and male infertility, including Ma et al. (2022) and Han et al. (2018).

**Fig. 1:**
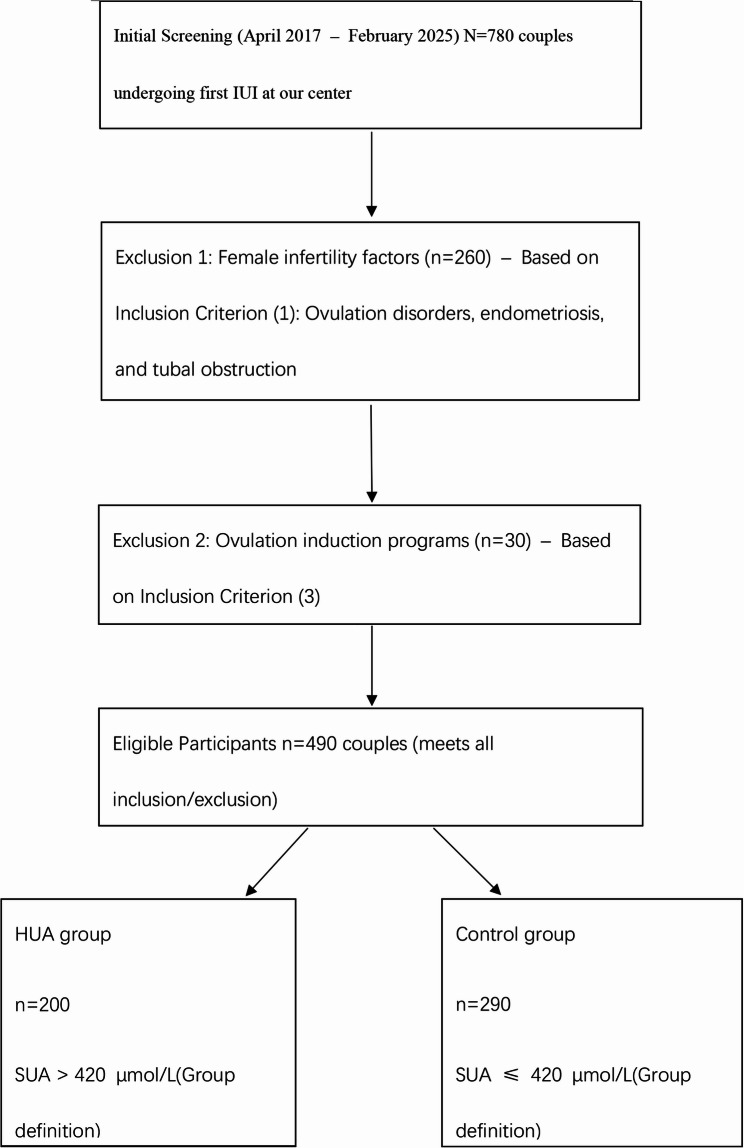
Legend: This flowchart illustrates the enrollment process of participants in the retrospective cohort study. A total of 780 couples undergoing their first intrauterine insemination (IUI, a common assisted reproductive technology for infertility treatment) cycle were initially screened between April 2017 and February 2025 (the study’s initial screening period). Couples were excluded if they met the following criteria: 260 couples with female infertility (including ovulation disorders, endometriosis, and tubal obstruction, which does not meet inclusion criterion ①) and 30 couples receiving ovulation induction (which does not meet inclusion criterion ③). Finally, 490 eligible couples were stratified into two groups based on male serum uric acid (SUA, a metabolic marker reflecting uric acid levels in blood) levels—with the grouping threshold referring to *Guideline for the Diagnosis and Management of Hyperuricemia and Gout in China (2019)* and *China Multi-Disciplinary Expert Consensus on Diagnosis and Treatment of Hyperuricemia and Related Diseases (2023 Edition)*: the control group (SUA ≤ 420 µmol/L, *n* = 290) and the hyperuricemia (HUA) group (SUA > 420 µmol/L, *n* = 200) Inclusion criteria: ① Male factor infertility or unexplained infertility (couples with female infertility are excluded); ② First IUI cycle; ③ Natural ovulatory cycle (couples receiving ovulation induction are excluded) Exclusion criteria: ① Genetic, congenital, endocrine, or immune disorders; varicocele; genital tract infections; ② Recent fever, exposure to high temperatures, or toxin exposure; ③ Testicular injury, history of mumps, or long-term medication use

The final grouping was as follows: The first IUI cycles of 490 male partners, including 200 cycles in the HUA group and 290 cycles in the control group, were included. The participant selection process is illustrated in Fig. 1.

### Post hoc power analysis for sample size

Given the retrospective cohort design, post hoc power analysis was conducted to verify the adequacy of the enrolled sample size using G*Power 3.1 software (Heinrich-Heine-Universität Düsseldorf, Germany). The primary endpoint was defined as the between-group difference in ΔPR% (change in percentage of progressively motile sperm) before and following DGC, and the analysis was based on the actual study data with the following parameters:

- Observed effect size (d): 0.21 (calculated from ΔPR% data: HUA group 52.34% ± 10.62% vs. control group 50.29% ± 11.02%);
- Significance level (α): 0.05 (two-tailed);Significance level (α): 0.05 (two-tailed);
- Sample size: 290 in the control group, 200 in the HUA group;
- Allocation ratio (control: HUA): 1.45:1 (consistent with the actual retrospective enrollment distribution).

The post hoc power analysis showed that the statistical power (1-β) for detecting the observed ΔPR% difference was 0.83, exceeding the conventional threshold of 0.80, indicating the sample size was sufficient to reliably detect the significant difference in the primary endpoint. For the secondary endpoint (clinical pregnancy rate), the observed effect size was smaller (d = 0.06), and the corresponding statistical power was 0.22, which may explain the lack of significant between-group difference.

## Methods

### Measurement of serum uric acid levels

The participants fasted for 8–12 h before blood samples were collected. A total of 4 mL of cubital venous blood was collected in the morning. The serum was centrifuged, separated, and stored in a −80 °C freezer until further testing. The SUA level was determined via the uricase method (CV ≤ 4.0%, relative deviation ≤ 10.0%).

### Sperm collection and analysis

After 3–7 days of abstinence, semen samples were collected via masturbation. Following placement in a 37 °C, 6% CO₂ incubator for 15 min, the specimens were observed every 5 min to assess for complete liquefaction. After complete liquefaction, the semen specimen was mixed thoroughly with a Pasteur pipette, and 10 µl of semen was immediately aspirated and placed on a Makler counting chamber (Sefi Medical Instruments Ltd.) for 1 min. Basic semen analysis was performed manually under a light microscope according to the *WHO Laboratory Manual for the Examination and Processing of Human Semen (5th Edition)* [16].

To ensure the accuracy and consistency of results across operators:

1. All laboratory personnel received standardized training on WHO semen analysis protocols and passed proficiency testing prior to performing procedures;
2. For each specimen, sperm motility and concentration were independently assessed by two trained operators, with a third operator consulted to resolve discrepancies (coefficient of variation < 5% for consistent results);
3. Monthly calibration was performed using commercial quality control specimens (Bio-Rad, Hercules, USA) to verify the reliability of manual counting.

The following parameters were measured: semen volume (mL), sperm concentration (×10⁶/mL), percentage of progressively motile sperm (PR%), percentage of nonprogressively motile sperm (NP%), percentage of immotile sperm (IM%), and total progressively motile sperm count (TPMSC).

### Density gradient centrifugation

All reagents were purchased from Vitrolife (Sweden). The density gradient centrifugation procedure comprised three consecutive steps:

1. Gradient solution preparation: Prepared using SpermGrad™ (Catalog No.: 10099, Vitrolife, Sweden) with a standard 90% bottom layer (1.5 mL) and 45% upper layer (1.5 mL). Thoroughly mixed semen samples (1–2 mL) were slowly added to the upper layer of the gradient solution (avoiding mixing), balanced, and centrifuged at 300 × g for 15 min. For large-volume samples, aliquots were centrifuged separately, and the bottom precipitates were pooled.
2. Sperm cluster washing: The collected precipitated sperm was resuspended in 3 mL of SpermRinse™ (Catalog No.: 10101) and centrifuged at 300 × g for 5 min, after which the supernatant was discarded.
3. Final resuspension: The precipitated sperm was resuspended in 0.3–0.5 mL of G-IVF PLUS™ (Catalog No.: 10136) and mixed well for subsequent analysis.

A total of 10 µL of semen was aspirated, placed on a counting plate for 1 min, and observed under a microscope. Following DGC processing, the semen volume (mL), sperm concentration (×10⁶/mL), PR%, NP%, IM%, and TPMSC were recorded.

### Calculation of semen parameter changes following density gradient centrifugation

The changes in semen parameters before (T0) and following DGC processing (T1) were calculated as the difference between post-treatment (T1) and pre-treatment (T0) values. The specific formulas are as follows:

$$\begin{aligned} \triangle\mathrm{PR}\;\left(\%\right)\;=&\;\mathrm P{\mathrm R}_-\mathrm T1\;-\;\mathrm P{\mathrm R}_-\mathrm T0\;\left(\triangle\mathrm{PR}:\;\mathrm{Change}\;\mathrm{in}\; \right. \\& \left. \mathrm{percentage}\;\mathrm{of}\;\mathrm{progressively}\;\mathrm{motile}\;\mathrm{sperm}\right) \end{aligned}$$

$$\begin{aligned} \triangle\mathrm{NP}\;\left(\%\right)\;=&\;{\mathrm{NP}}_-\mathrm T1\;-\;{\mathrm{NP}}_-\mathrm T0\;\left(\triangle\mathrm{NP}:\;\mathrm{Change}\;\mathrm{in}\;\right. \\& \left. \mathrm{percentage}\;\mathrm{of}\;\mathrm{nonprogressively}\;\mathrm{motile}\;\mathrm{sperm}\right) \end{aligned}$$

$$\begin{aligned} \triangle\mathrm{IM}\;\left(\%\right)\;=&\;{\mathrm{IM}}_-\mathrm T1\;-\;{\mathrm{IM}}_-\mathrm T0\;\left(\triangle\mathrm{IM}:\;C\mathrm{hange}\;\mathrm{in}\; \right. \\& \left. \mathrm{percentage}\;\mathrm{of}\;\mathrm{immotile}\;\mathrm{sperm}\right) \end{aligned}$$

$$\begin{aligned} \triangle\mathrm{TPMSC}\;=&\;{\mathrm{TPMSC}}_-\mathrm T1\;-\;{\mathrm{TPMSC}}_-\mathrm T0\;\\& \left(\triangle\mathrm{TPMSC}:\; \mathrm{Change}\;\mathrm{in}\;\mathrm{total}\; \right.\\& \left. \mathrm{progressively}\;\mathrm{motile}\;\mathrm{sperm}\;\mathrm{count}\right) \end{aligned}$$

$$\begin{aligned} &\triangle\mathrm{Semen}\;\mathrm{volume}\;\left(\mathrm{mL}\right)\;\\&=\;\mathrm{Semen}\;{\mathrm{volume}}_-\mathrm T1\;-\;\mathrm{Semen}\;{\mathrm{volume}}_-\mathrm T0\;\\&\left (\triangle\mathrm{Semen}\;\mathrm{volume}:\:\mathrm{Change}\;\mathrm{in}\;\mathrm{semen}\;\mathrm{volume}\right) \end{aligned}$$

$$\begin{aligned} &\triangle\mathrm{Sperm}\;\mathrm{concentration}\;\left(\times10^6/\mathrm{mL}\right)\\&=\;\mathrm{Sperm}\;{\mathrm{concentration}}_-\mathrm T1\;-\;\mathrm{Sperm}\;{\mathrm{concentration}}_-\mathrm T0\;\\&\left(\triangle\mathrm{Sperm}\;\mathrm{concentration}:\:\mathrm{Change}\;\mathrm{in}\;\mathrm{sperm}\;\mathrm{concentration}\right) \end{aligned}$$

### Intrauterine insemination cycles and pregnancy outcomes

#### Intrauterine insemination procedure

At 36 h after human chorionic gonadotropin (hCG) injection, the DGC-processed sperm suspension (0.5 mL) was injected into the uterine cavity.

#### Confirmation of pregnancy

Preliminary pregnancy screening was conducted 14 days after IUI using either a qualitative urine β-human chorionic gonadotropin (β-hCG) test or a quantitative serum β-hCG test (positive threshold: serum β-hCG > 25 IU/L). Participants with positive screening results underwent transvaginal B-type ultrasound at 5 weeks post-IUI (6 weeks of gestation) for further confirmation: Clinical pregnancy was diagnosed if a gestational sac (with or without a yolk sac) was identified in the uterine cavity;

Biochemical pregnancy was diagnosed if β-hCG levels were elevated (positive screening) but no gestational sac was detected on ultrasound, in line with the definition specified in The International Glossary on Infertility and Fertility Care [17].

### Statistical analysis

SPSS 26.0 software was used for all statistical analyses.

- **Normally distributed measurement data** (e.g., ΔPR%, pretreatment PR%) are expressed as the means ± standard deviations, with intergroup comparisons performed using an independent sample t test.
- **Nonnormally distributed measurement data** (e.g., age, sperm concentration, TPMSC) are expressed as medians (interquartile ranges), with intergroup comparisons performed using the Mann‒Whitney U test.
- **Categorical variables** (e.g., pregnancy outcomes, infertility type) are expressed as frequencies and percentages. Intergroup comparisons for categorical variables (e.g., pregnancy outcomes) were performed using the χ² test; additionally, binary logistic regression analysis was used to calculate unadjusted odds ratios (OR) and 95% confidence intervals (95% CI) for clinical pregnancy outcomes (with the control group as the reference).

The significance threshold was set at *P* < 0.05. Post hoc power analysis was conducted to verify the adequacy of the sample size for both the primary endpoint (ΔPR% difference) and secondary endpoint (clinical pregnancy rate), as detailed in Sect. 2.3.

Regarding multiple testing correction: For baseline intergroup comparisons (including PR%, sperm concentration, and other semen parameters), we applied the Bonferroni correction to control for Type I errors. The uncorrected P-value for baseline PR% difference was 0.038, and the corrected P-value was 0.040 (adjusted for 3 key baseline semen parameters: PR%, sperm concentration, TPMSC). This confirms that the marginal significance of the baseline PR% difference (corrected *P* = 0.040) remains valid after accounting for multiple comparisons.

## Results

### Baseline characteristics

No significant differences were observed in baseline characteristics—including age, infertility duration, and infertility type—between the two groups (Table 1).

**Table 1:** Comparison of baseline characteristics between the HUA group (SUA > 420 µmol/L) and control group (SUA ≤ 420 µmol/L)

Variable	Control group (*n* = 290)	HUA group (*n* = 200)	*P*
Average age of the male partner (years)	31.00 (29.00,34.00)	31.00 (29.00,34.00)	0.628
Average age of the female partner (years)	30.00 (28.00,33.00)	30.00 (28.00,33.00)	0.672
Types of female infertility			0.334
Primary infertility	111 (38.3%)	68 (34.0%)	
Secondary infertility	179 (61.7%)	132 (66.0%)	
Duration of female infertility (years)	2.00 (2.00,3.00)	2.00 (2.00,3.75)	0.994
Number of days of abstinence (days)	4.00 (3.00,5.00)	3.00 (3.00,5.00)	0.952

This table presents baseline clinical characteristics across the two groups, including continuous variables (male age, female age, infertility duration, abstinence days) and categorical variables (female infertility type: primary infertility = no prior pregnancy, secondary infertility = prior pregnancy). Due to non-normal distribution of all continuous variables, they are expressed as medians (interquartile ranges, IQR, a measure of data dispersion); categorical variables are presented as frequencies (percentages). Intergroup comparisons were performed using the Mann-Whitney U test (for non-normal continuous variables) and the χ² test (for categorical variables). Statistical significance was defined as a two-tailed P-value < 0.05

*Abbreviations*: *HUA *Hyperuricemia (SUA > 420 µmol/L, per Chinese clinical guidelines), *SUA *Serum uric acid, *IQR *Interquartile range

### Semen quality and conditions following density gradient centrifugation processing

The semen volume (mL), sperm concentration (×10⁶/mL), PR%, NP%, IM%, and TPMSC on the IUI day were compared between the two groups. Following DGC processing, the semen volume (mL), sperm concentration (×10⁶/mL), PR%, NP%, IM%, and TPMSC were measured, and the differences between the two groups after DGC processing were analyzed.

At baseline, PR% was significantly lower in the HUA group than in the control group (39.55% ± 11.29% vs. 41.76% ± 11.89%, *P* = 0.040) (Table 2).

**Table 2:** Comparison of pretreatment (pre-DGC) semen parameters between the HUA group (SUA > 420 µmol/L) and control group (SUA ≤ 420 µmol/L)

Variable	Control group (*n* = 290)	HUA group (*n* = 200)	*P*
Pretreatment volume (mL)	3.00 (2.08, 3.80)	3.00 (2.10, 3.50)	0.211
Pretreatment concentration (10^6^/mL)	56.50 (42.00, 82.00)	58.00 (43.00, 79.50)	0.819
Pretreatment PR(%)	41.76 ± 11.89	39.55 ± 11.29	0.040
Pretreatment NP (%)	9.00 (6.75, 15.00)	9.00 (7.00, 14.00)	0.519
Pretreatment IM (%)	46.00 (38.00, 57.00)	48.00 (41.00, 58.00)	0.039
Pretreatment TPMSC (10^6^)	66.50 (41.44, 95.92)	61.78 (37.64,89.67)	0.230
Liquefaction time (min)	30.00 (20.00,30.00)	30.00 (20.00,30.00)	0.795

This table presents pretreatment semen parameters (volume = semen amount, concentration = sperm count per mL, PR% = Percentage of progressively motile sperm [sperm moving forward actively], NP% = Percentage of nonprogressively motile sperm [sperm moving without forward direction], IM% = Percentage of immotile sperm, TPMSC = Total progressively motile sperm count [total number of actively forward-moving sperm], liquefaction time = time for semen to change from gel to liquid) across the two groups. “Pretreatment” refers to semen parameters measured before density gradient centrifugation (DGC, a common semen processing method to select high-quality sperm for IUI). Data for normally distributed variables (pretreatment PR%) are expressed as means ± standard deviations (SD, a measure of data dispersion); data for non-normally distributed variables (pretreatment volume, concentration, NP%, IM%, TPMSC, liquefaction time) are presented as medians (interquartile ranges, IQR). Intergroup comparisons were performed using the independent samples t-test (for normally distributed continuous data) and the Mann-Whitney U test (for non-normally distributed continuous data). Statistical significance was defined as a two-tailed P-value < 0.05

*Abbreviations*: *HUA *Hyperuricemia, *SUA *Serum uric acid, *PR% *Percentage of progressively motile sperm, *NP% *Percentage of nonprogressively motile sperm, *IM% *Percentage of immotile sperm, *TPMSC *Total progressively motile sperm count, *DGC *Density gradient centrifugation, *SD *Standard deviation, *IQR *Interquartile range

Following DGC processing, PR% exceeded 90% in both groups, with no statistically significant difference (HUA group: 92% [90%, 95%] vs. control group: 93% [90%, 94%], *P* = 0.557) (Table 3).

**Table 3:** Comparison of posttreatment (post-DGC) semen parameters between the HUA group (SUA > 420 µmol/L) and control group (SUA ≤ 420 µmol/L)

Variable	Control group (*n* = 290)	HUA group (*n* = 200)	*P*
Posttreatment volume (mL)	0.50 (0.50, 0.50)	0.50 (0.50, 0.50)	0.761
Posttreatment concentration (10^6^/mL)	68.00 (44.50, 95.00)	63.00 (40.00, 88.75)	0.152
Posttreatment PR (%)	93.00 (90.00, 94.00)	92.00 (90.00, 95.00)	0.557
Posttreatment NP (%)	3.00 (2.00, 4.00)	3.00 (2.00, 4.00)	0.904
Posttreatment IM (%)	4.00 (3.00, 5.00)	4.00 (3.00, 6.00)	0.651
Posttreatment TPMSC (10^6^)	31.51 (19.77, 41.84)	28.83 (18.10, 40.89)	0.182

This table presents posttreatment semen parameters (volume = semen amount, concentration = sperm count per mL, PR% = Percentage of progressively motile sperm [actively forward-moving], NP% = Percentage of nonprogressively motile sperm [non-forward-moving], IM% = Percentage of immotile sperm, TPMSC = Total progressively motile sperm count [total actively forward-moving sperm]) across the two groups. “Posttreatment” refers to semen parameters measured following density gradient centrifugation (DGC, a semen processing method to select high-quality sperm for IUI). Due to the non-normal distribution of all semen parameters, data are expressed as medians (interquartile ranges, IQR, a measure of data dispersion). Intergroup comparisons were performed using the Mann-Whitney U test (for non-normally distributed continuous variables). Statistical significance was defined as a two-tailed P-value < 0.05

*Abbreviations*: *HUA *Hyperuricemia, *SUA *Serum uric acid, *PR% *Percentage of progressively motile sperm, *NP% *Percentage of nonprogressively motile sperm, *IM% *Percentage of immotile sperm, *TPMSC *Total progressively motile sperm count, *DGC *Density gradient centrifugation, *IQR *Interquartile range

### Key findings

The effect of DGC on the ΔPR% in the HUA group was significantly greater than that in the control group (ΔPR%: 52.34% ± 10.62% [HUA] vs. 50.29% ± 11.02% [control], *P* = 0.040) (Table 4).

**Table 4:** Comparison of changes in semen parameters before and following density gradient centrifugation (DGC) between the HUA group (SUA > 420 µmol/L) and control group (SUA ≤ 420 µmol/L)

Variable	Control group (*n* = 290)	HUA group (*n* = 200)	*P*
ΔSperm volume (mL)	−2.50 (−3.30, −1.60)	−2.50 (−3.00, −1.60)	0.207
ΔSperm concentration (10^6^/mL)	9.40 ± 28.21	5.41 ± 32.40	0.148
ΔPR (%)	50.29 ± 11.02	52.34 ± 10.62	0.040
ΔNP (%)	−6.00 (−11.00, −4.00)	−6.00 (−10.00, −4.00)	0.505
ΔIM (%)	−5.00 (−10.00, −3.00)	−5.00 (−8.75, −3.00)	0.424
ΔTPMSC (10^6^)	−35.28 (−54.23, −20.87)	−31.76 (−53.71, −18.69)	0.243

Δ indicates the difference in values of sperm parameters before (pretreatment) and after density gradient centrifugation (DGC, a semen processing method to select high-quality sperm for IUI) (posttreatment), calculated as “posttreatment value – pretreatment value”. This table presents these Δ values (e.g., ΔPR% = post-DGC PR% – pre-DGC PR%) across the two groups. Data for normally distributed variables (Δsperm concentration, ΔPR% = change in percentage of progressively motile sperm) are presented as means ± standard deviations (SD, a measure of data dispersion); data for non-normally distributed variables (Δsperm volume, ΔNP% = change in nonprogressively motile sperm percentage, ΔIM% = change in immotile sperm percentage, ΔTPMSC = change in total progressively motile sperm count) are presented as medians (interquartile ranges, IQR). Intergroup comparisons were performed using the independent samples t-test (for normally distributed continuous data) and the Mann-Whitney U test (for non-normally distributed continuous data). Statistical significance was defined as a two-tailed P-value < 0.05

*Abbreviations*: *HUA *Hyperuricemia, *SUA *Serum uric acid, *PR% *Percentage of progressively motile sperm, *NP% *Percentage of nonprogressively motile sperm, *IM% *Percentage of immotile sperm, *TPMSC *Total progressively motile sperm count, *DGC *Density gradient centrifugation, *SD *Standard deviation, *IQR *Interquartile range

No significant difference was noted in the clinical pregnancy rate between the two groups (11.0% vs. 13.4%, χ² = 1.432, *P* = 0.230). Binary logistic regression analysis further demonstrated that the HUA group had no statistically significant association with clinical pregnancy outcomes compared with the control group (unadjusted OR = 0.80, 95% CI: 0.46–1.39, *P* = 0.423) (Table 5). Similarly, no significant difference was observed in the biochemical pregnancy rate between the two groups (1.4% vs. 3.5%, *P* = 0.102).

**Table 5:** Comparison of intrauterine insemination (IUI) pregnancy outcomes between the HUA group (SUA > 420 µmol/L) and control group (SUA ≤ 420 µmol/L)

Variable	Control group (*n* = 290)	HUA group (*n* = 200)	Unadjusted OR (95% CI)	*P*
Pregnancy outcomes	-	-	-	0.230
Clinical pregnancy	39 (13.4%)	22 (11.0%)	0.80 (0.46–1.39)	0.423
Biochemical pregnancy	4 (1.4%)	7 (3.5%)	2.59 (0.82–8.19)	0.102
Not pregnant	247 (85.2%)	171 (85.5%)		

This table presents the distribution of categorical pregnancy outcomes (clinical pregnancy, biochemical pregnancy, non-pregnancy) in the hyperuricemia (HUA) group and the control group. Outcome definitions: Clinical pregnancy = visualization of a gestational sac (a sign of early pregnancy) on transvaginal B-ultrasound at 5 weeks after IUI; Biochemical pregnancy = positive β-human chorionic gonadotrophin (β-HCG, a hormone produced during pregnancy) detection (serum β-HCG > 25 IU/L) without a visible gestational sac on B-ultrasound; Non-pregnancy = negative β-HCG and no gestational sac. Data are expressed as frequencies (percentages). Intergroup comparisons for pregnancy outcomes were performed using the χ² test (for categorical variables). Additionally, binary logistic regression analysis was conducted to calculate the odds ratio (OR, a measure of association between HUA and pregnancy: OR < 1 = lower pregnancy odds in HUA group) and 95% confidence interval (CI, a range of values for OR with 95% certainty), with the control group set as the reference. Statistical significance was defined as a two-tailed P-value < 0.05

*Abbreviations*: *HUA *Hyperuricemia, *SUA *Serum uric acid, *β-HCG *β-human chorionic gonadotrophin (pregnancy-related hormone), *IUI *Intrauterine insemination (assisted reproductive technology), *OR *Odds ratio, *CI *Confidence interval

To further verify the robustness of pregnancy outcome results, we performed adjusted binary logistic regression analysis, incorporating female age (continuous variable) and female infertility type (categorical variable: primary infertility/secondary infertility) as confounding factors. The results showed that after adjustment, the association between HUA and clinical pregnancy remained non-significant (adjusted OR = 0.78, 95% CI: 0.44–1.37, *P* = 0.386), consistent with the unadjusted result (unadjusted OR = 0.80, 95% CI: 0.46–1.39, *P* = 0.423). This confirms that the non-significant difference in clinical pregnancy rate between groups is robust to potential confounding by female age and infertility type.

## Discussion

To the best of our knowledge, this is the first study to demonstrate that DGC significantly improves sperm motility in patients with HUA (ΔPR%: HUA group 52.34% ± 10.62% vs. control group 50.29% ± 11.02%, *P* = 0.040), offering a novel strategy to optimize ART for patients with HUA-associated male infertility. This finding not only validates the biological screening performance of DGC but also suggests that the alleviation of oxidative stress may be a key therapeutic strategy for improving semen quality.

The impairment of sperm quality by HUA involves multiple pathological pathways. First, oxidative stress plays a central role: studies have confirmed that oxidative stress can directly damage testicular cells and sperm, resulting in decreased semen quality and male fertility [18]. Patients with HUA exhibit reduced antioxidant capacity, as evidenced by lower superoxide dismutase (SOD) and catalase (CAT) activity in seminal plasma and testicular tissue, elevated malondialdehyde (MDA) levels, and decreased concentrations of key antioxidants including ascorbic acid and uric acid (UA) [2, 3, 5]. Additionally, elevated homocysteine (Hcy) levels in patients with HUA induce oxidative stress, damaging sperm membranes and triggering apoptosis [6, 19]. Second, HUA inhibits gonadal axis function: high UA levels are associated with reduced serum testosterone, which is critical for spermatogenesis, sperm maturation, and capacitation [20]. Third, HUA impairs accessory sex gland function: decreased α-glucosidase in seminal plasma (secreted by epididymal epithelial cells) leads to insufficient glucose supply for sperm motility [3, 5].

Notably, while the abstract references “mechanistic insights” into DGC’s effect on HUA-associated sperm motility, it is critical to clarify that direct measurement of mechanistic markers (e.g., oxidative stress mediators, metabolic factors such as reactive oxygen species [ROS], MDA, SOD) was not conducted in this retrospective study. All mechanistic inferences—including DGC’s role in scavenging ROS and optimizing energy supply—are derived from alignment with prior experimental studies, rather than primary data from the current cohort. Even so, our findings remain consistent with existing evidence linking HUA to oxidative stress and its subsequent impacts on sperm motility:

(1) Prior studies confirmed that patients with HUA exhibit reduced antioxidant capacity (lower SOD/CAT activity, higher MDA levels) and elevated oxidative stress mediators (e.g., homocysteine), which directly damage sperm membrane integrity and energy metabolism;

(2) Our data showed that the HUA group had a significantly lower baseline PR% (39.55% ± 11.29% vs. 41.76% ± 11.89%, *P* = 0.040) and higher IM% (48.00% vs. 46.00%, *P* = 0.039)—these differences are consistent with the pathological effect of oxidative stress on sperm motility;

(3) The greater improvement in ΔPR% in the HUA group following DGC (52.34% ± 10.62% vs. 50.29% ± 11.02%, *P* = 0.040) correlates with DGC’s known capacity to remove ROS and pathological metabolites [21], suggesting DGC may specifically counteract HUA-induced oxidative damage.

These results collectively support that oxidative stress is a key pathway linking HUA to sperm motility impairment, even without direct measurement of oxidative stress markers.

In vitro DGC processing effectively alleviates HUA-associated sperm damage through multiple mechanisms. Takeshima et al. demonstrated that DGC separates motile sperm from immotile ones and removes ROS, reducing oxidative stress damage. Additionally, DGC eliminates debris, white blood cells, and non-sperm cells, minimizing the adverse effects of seminal plasma toxins [22]. Moreover, the culture medium used in DGC (containing pyruvate, lactic acid, and glucose) provides energy substrates for sperm via glycolysis, oxidative phosphorylation (OXPHOS), and the tricarboxylic acid (TCA) cycle, compensating for energy supply defects caused by reduced α-glucosidase [23, 24].

Although DGC significantly improved sperm motility in the HUA group, there was no difference in the clinical pregnancy rate between the two groups (11.0% vs. 13.4%, *P* = 0.230). Notably, the 2% difference in ΔPR% (52.34% ± 10.62% vs. 50.29% ± 11.02%, *P* = 0.040) requires careful interpretation of clinical relevance beyond statistical significance.

Importantly, this finding also represents the core innovation of our study: To the best of our knowledge, this is the first to verify DGC’s therapeutic effect on HUA-specific sperm damage. Existing studies on DGC have focused primarily on method comparisons and efficacy evaluations in the general infertile population, with no reports targeting patients with HUA (who have metabolic abnormalities)—thus addressing this key knowledge gap.

This ΔPR% difference reflects targeted clinical value for patients with HUA: first, the HUA group had a significantly lower baseline PR% (39.55% vs. 41.76%, *P* = 0.040), indicating inherent sperm motility impairment due to hyperuricemia. The greater ΔPR% improvement in this group suggests DGC specifically reverses HUA-associated damage, rather than a trivial numerical difference—this specificity validates DGC as a targeted semen processing method for metabolically abnormal patients. Second, the difference supports the proposed mechanisms: it aligns with DGC’s capacity to scavenge ROS and supply energy substrates, directly addressing HUA’s pathological weaknesses (oxidative stress and energy deficiency). Third, it informs individualized ART practice: the finding suggests DGC may be preferred over conventional washing for patients with HUA, providing a basis to optimize laboratory protocols.

The lack of improvement in clinical pregnancy rate, despite DGC’s specific benefit for sperm motility in HUA patients, may be attributed to four interrelated factors, which together highlight the complexity of translating improved semen parameters to clinical outcomes. First, post-DGC PR% in both groups exceeded 90% (HUA: 92%, control: 93%), reaching a “clinical efficacy threshold”—beyond this point, other determinants of pregnancy are likely to dominate. These include sperm DNA integrity (DNA Fragmentation Index, DFI); HUA-induced oxidative stress may damage sperm DNA independently of motility, even after DGC processing [3, 19], which could undermine fertilization potential despite enhanced motility. Second, sperm motility is only one of multiple prerequisites for fertilization success. Even though DGC removes ROS from seminal plasma and improves motility in vitro, HUA-induced oxidative stress may have already caused persistent impairments to critical sperm functional competencies (e.g., capacitation, acrosome reaction, sperm-oocyte binding) before semen collection [18, 23]. These defects could act as “rate-limiting steps” in fertilization, ultimately overriding the benefit of enhanced sperm motility after DGC. Third, suboptimal female reproductive factors may have masked the potential benefit of improved sperm quality: high missing rates of female ovarian reserve (Anti-Müllerian Hormone, AMH, 45.7%) and unrecorded endometrial thickness in early retrospective data prevent us from ruling out confounding by factors like diminished ovarian reserve or thin endometrium—both of which are well-recognized key determinants of IUI success. Fourth, the sample size of the HUA subgroup (*n* = 200) may limit the ability to detect small but clinically meaningful differences in pregnancy rates, particularly for secondary endpoints (further details on statistical power are provided in the Limitations section).

### Limitations of the study

This study has several limitations that should be acknowledged. First, as a retrospective cohort study, it is inherently subject to selection bias and residual confounding. Regarding selection bias: participant inclusion relied on complete clinical records (e.g., serum uric acid levels, semen parameters, pregnancy outcomes) from our center’s database; patients with incomplete data were excluded, which may have skewed the sample toward individuals with more regular clinical follow-up and limited generalizability to the broader HUA-associated infertility population. Regarding residual confounding: although we balanced key baseline characteristics (e.g., female age, infertility type) via strict inclusion/exclusion criteria (Table 1), critical confounders could not be fully addressed—including female age-related declines in oocyte quality (subtle variations in oocyte competence may have influenced outcomes), diminished ovarian reserve (exacerbated by 45.7% AMH data missing rate), and potential inconsistencies in ovulation monitoring (minor variations in hCG injection timing or monitoring frequency in early records).

Second, the extended study period (April 2017–February 2025 [8 years]) may introduce additional biases: changes in clinical IUI protocols (e.g., hCG injection timing, uterine catheter selection) or laboratory personnel turnover could lead to inconsistencies in semen parameter analysis (e.g., subjective PR% assessment) or DGC processing (e.g., gradient solution preparation accuracy). Although we mitigated this via standardized operating procedures (SOPs), quarterly staff training, and annual equipment calibration, residual variability cannot be fully excluded.

Third, post hoc power analysis showed the sample size was sufficient for the primary endpoint (ΔPR% difference, 1-β = 0.83), but statistical power for the secondary endpoint (clinical pregnancy rate) was only 0.22 due to a smaller observed effect size (d = 0.06). This low power may have prevented detection of potential small differences in pregnancy outcomes.

Fourth, we did not measure sperm DFI—a key parameter reflecting sperm genetic integrity. This omission limits our ability to explain why improved motility did not translate to higher pregnancy rates, as HUA may impair sperm DNA independently of motility [3, 19].

Fifth, we did not evaluate key sperm functional parameters essential for fertilization (e.g., capacitation, acrosome reaction). These processes are sensitive to oxidative stress and metabolic disturbances [18, 23]—pathological features of HUA—and this gap limits a comprehensive evaluation of DGC’s therapeutic value, as unassessed functional defects may have affected outcomes.

Sixth, we were unable to perform multivariate logistic regression to adjust for confounding factors (e.g., female body mass index [BMI], endometrial thickness) affecting pregnancy outcomes, due to high missing rates (female BMI: 38.2%). While baseline balancing mitigated residual confounding (Table 1), unmeasured or missing factors still cannot be ruled out, and pregnancy rate results should be interpreted cautiously.

Seventh, we did not collect male BMI data—a potential confounder for semen quality. Abnormal male BMI can reduce semen parameters and alter seminal plasma metabolites [25], which may interact with HUA-induced metabolic dysregulation to affect sperm motility. This gap is attributed to retrospective design (male BMI was not routinely recorded); partial data missingness (> 40%) posed selection bias risk, so it was excluded from analysis.

Eighth, we did not directly measure seminal plasma oxidative stress markers (e.g., ROS, MDA, SOD), which limits direct verification of the “HUA-oxidative stress-sperm damage” pathway. The correlation between HUA and oxidative stress was inferred from existing literature and indirect semen parameter differences (e.g., lower baseline PR% in the HUA group), rather than direct experimental evidence. Future prospective studies should include these markers to confirm the pathway.

## Conclusion

To the best of our knowledge, this study is the first to confirm that DGC specifically improves impaired sperm motility in patients with HUA: the baseline PR% in the HUA group was significantly lower than that in the control group (39.55% vs. 41.76%, *P* = 0.040), while the increase in PR% (ΔPR%) following DGC was significantly greater in the HUA group (52.34% ± 10.62% vs. 50.29% ± 11.02%, *P* = 0.040), with both groups achieving PR% values exceeding 90% post-treatment. Notably, this improvement in motility did not translate to a higher clinical pregnancy rate (11.0% vs. 13.4%, *P* = 0.230).

These findings have important implications for clinical practice and research. Clinically, they validate DGC as a targeted semen preparation method for HUA-associated male infertility, providing a basis for individualized ART strategies. From a biological perspective, the observed efficacy of DGC in patients with HUA may correlate with its capacity to remove ROS and pathological metabolites (alleviating oxidative stress) and supply energy substrates (compensating for HUA-induced α-glucosidase deficiency), though this is inferred from prior mechanistic studies [21, 23, 24] rather than from direct evidence in our observational data. However, the lack of difference in pregnancy outcomes suggests HUA causes systemic reproductive impairment beyond motility defects, highlighting the need for multitargeted interventions.

For future research, three strategies may further optimize the clinical value of DGC in HUA-associated male infertility and address the gap between improved sperm motility and unaltered pregnancy outcomes. First, functionalizing DGC gradient solutions with ROS scavengers (e.g., glutathione) could enhance the removal of residual oxidative stress mediators in seminal plasma, potentially reversing HUA-induced sperm functional defects beyond motility (e.g., capacitation or acrosome reaction impairments). Second, stratifying patients with HUA by baseline PR% (per POSEIDON criteria) may enable personalized DGC application, improving both efficacy and cost-effectiveness by targeting subgroups most likely to benefit. Third, extending follow-up duration to track live birth rates (rather than just clinical pregnancy) and incorporating additional outcome measures—such as in vitro sperm-oocyte binding efficiency or time-to-pregnancy—could provide more comprehensive insights into DGC’s translational impact. Clinical pregnancy does not always culminate in live birth (e.g., due to early miscarriage), and these expanded metrics would help validate whether DGC’s biological effect on sperm motility translates to meaningful clinical benefits. These approaches, while beyond the scope of the current retrospective study, would strengthen the evidence base for DGC in metabolically abnormal infertile populations and guide further optimization of ART strategies for HUA patients.

Overall, this study fills the gap in research on DGC in metabolically abnormal infertile populations and offers practical guidance for improving ART outcomes in patients with HUA.

## Data Availability

The de-identified participant-level data (including semen parameters and pregnancy outcomes) analyzed in this retrospective cohort study are available from the corresponding author upon reasonable request, in compliance with the institutional data protection policies. Data sharing is contingent upon approval by the Institutional Review Board of the Affiliated Hospital of Putian University (Approval No. 2025171) to ensure compliance with data protection regulations.

## Abbreviations

HUA: Hyperuricemia
DGC: Density Gradient Centrifugation
IUI: Intrauterine Insemination
PR%: Percentage of Progressively Motile Sperm
NP%: Percentage of Nonprogressively Motile Sperm
IM%: Percentage of Immotile Sperm
TPMSC: Total Progressively Motile Sperm Count
SUA: Serum Uric Acid
ROS: Reactive Oxygen Species
SOD: Superoxide Dismutase
CAT: Catalase
MDA: Malondialdehyde
Hcy: Homocysteine
DFI: DNA Fragmentation Index
hCG: Human Chorionic Gonadotropin
OXPHOS: Oxidative Phosphorylation
TCA: Tricarboxylic Acid
ART: Assisted Reproductive Technology
POSEIDON: Patient-Oriented Strategies Encompassing Individualized Oocyte Number
BMI: Body Mass Index
AMH: Anti-Müllerian Hormone
SOP: Standard Operating Procedure
UA: Uric Acid
β-hCG: β-Human Chorionic Gonadotropin
